# Clinical and Radiological Aspects of Cough-induced Rib Fractures: A Case Report

**DOI:** 10.7759/cureus.6840

**Published:** 2020-02-01

**Authors:** Lucas S Bezerra, Gabrielle S Barbosa da Silva, Marcelo Antônio O Santos-Veloso, Mairton Amora Pinheiro Bosford, Ana Rita Marinho Ribeiro Carvalho

**Affiliations:** 1 Medicine, Maurício de Nassau University, Recife, BRA; 2 Internal Medicine, Hospital dos Servidores do Estado de Pernambuco, Recife, BRA; 3 Internal Medicine, Hospital dos Servidores do Estado, Recife, BRA; 4 Radiology, Hospital da Restauração, Recife, BRA

**Keywords:** cough, rib fracture, spontaneous fracture

## Abstract

Cough-induced rib fracture is a very rare condition, with a few cases described in the medical literature. This case report describes the case of a 77-year-old male patient with a history of chronic obstructive pulmonary disease (COPD) who presented with left eighth and ninth rib fractures after severe cough secondary to upper respiratory tract infection. The patient had a good clinical outcome, followed by conservative management. Conservative treatment is the first-choice approach in cases of daily activities limiting symptoms or complications. Cough-induced rib fracture should be remembered as a possible diagnosis, as diagnostic delays increase the risk of complications.

## Introduction

Cough is a physiological defense mechanism that protects the respiratory tract by clearing bronchial secretions and from foreign bodies. Nevertheless, cough has already been described to be associated with conditions such as urinary incontinence, syncope, pneumothorax, hernia, and rib fractures [1]. The most common etiology of costal fracture is blunt thoracic injury, whereas cough is an unusual cause [2-3].

This paper aims to report a case of a 77-year-old male presenting with left eighth and ninth rib fractures due to intense cough.

## Case presentation

A 77-year-old male presented to an ED complaining of productive cough, respiratory discomfort, and flu-like symptom for two weeks. A diagnosis of acute viral respiratory tract infection was done, and he was discharged with a prescription for nonsteroidal anti-inflammatory and decongestant.

The patient returned, one week after, complaining of the same symptoms and a new-onset severe abdominal pain. The pain was constant, intense (10/10), nonspecific, localized in the right upper quadrant, and not relieved with analgesic use. Moreover, there was no fever, rash, vomiting, or diarrhea. The patient was obese and had a history of arterial hypertension and chronic obstructive pulmonary disease (COPD). He denied any recent trauma.

His clinical examination revealed reduced air entry at the left lung base, an abnormal swelling on the left hypochondriac region, and a hard, painful, palpable mass lying immediately under, which was more prominent during inspiration.

**Figure 1:**
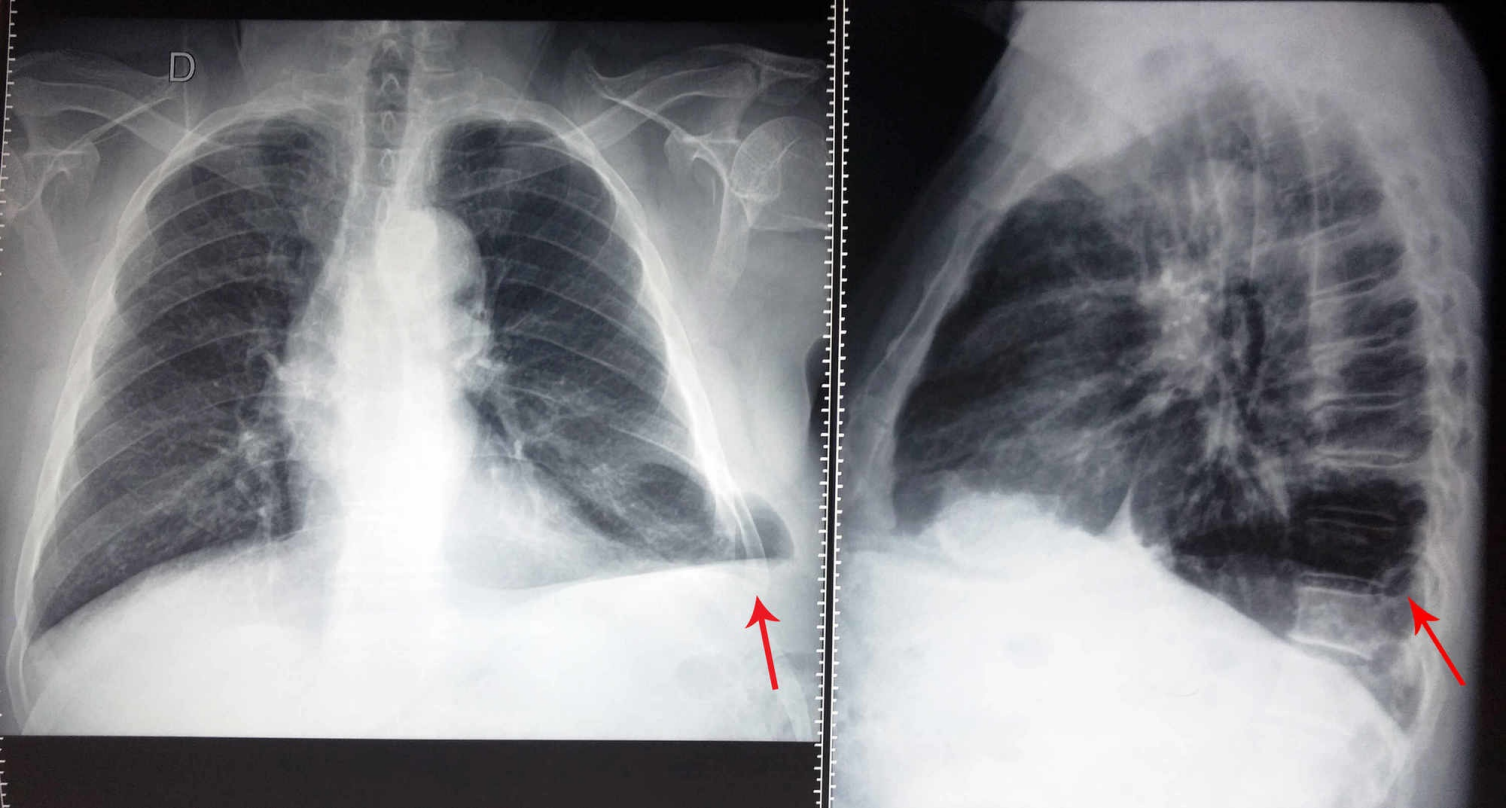
Chest X-ray posteroanterior (A) and lateral (B) view. The red arrow points an oval well-defined hypodensity in the left lower zone.

Laboratory studies revealed a mild leukocytosis (11.8 x 10³ leukocytes/mm³) without left shift. The chest X-ray posteroanterior view showed an oval well-defined hypodensity in the left lower zone (Figure 1).

**Figure 2:**
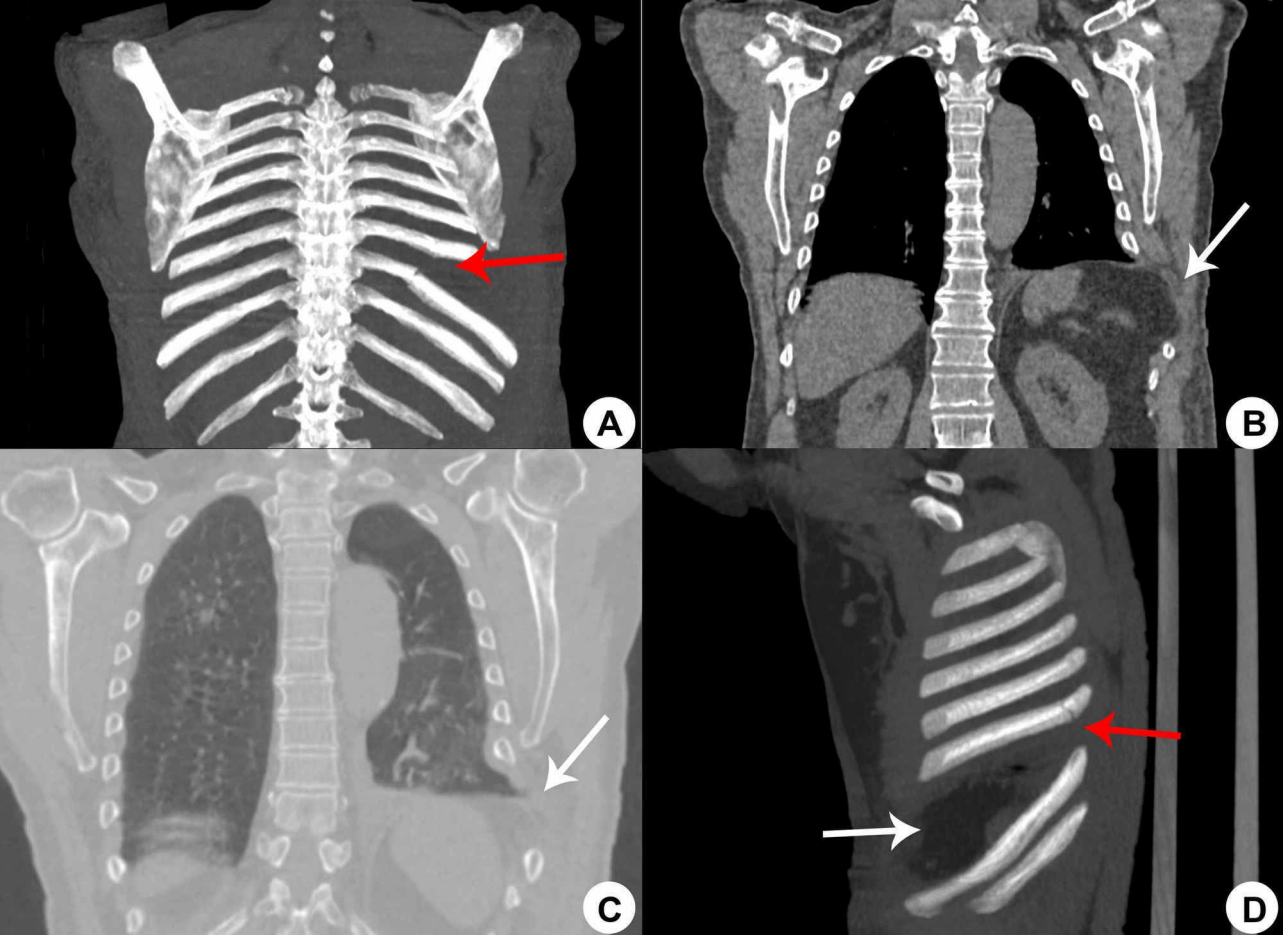
Computed axial tomographical image of the thorax. (A) Coronal reconstruction image showing ninth arch fracture and inferior rib dislocation. (B and C) Coronal view showing eight intercostal space enlargement, herniation of abdominal fat into the chest wall. (D) Sagittal reconstruction showing eighth arch fracture (red arrow), inferior rib dislocation and eight intercostal space enlargement (white arrow).

Due to the physical examination and X-ray findings, a thorax CT scan was performed and it revealed left eighth and ninth rib fractures, minimal pleural effusion on left hemithorax, ground-glass opacities, and atelectasis opacities in the lower left lobe (Figure 2).

Blood calcium measurements, serum protein electrophoresis, and other relevant studies performed to rule out secondary etiologies of pathological rib fracture were unchanged. Our thoracic surgical team adopted conservative treatment and the patient was discharged after pain control and supportive measures.

## Discussion

Cough-induced rib fracture usually is related to recurrent mechanical stress to the ribs, when the cough force is greater than the elastic limits of the ribs, which causes a fracture over the most vulnerable location [1].

Some conditions have been described as risk factors for cough-induced rib fractures, such as COPD, osteoporosis, rheumatic arthritis, and chronic corticosteroids use [2, 4-5]. In the case series reported by Mary Parks et al., 66.7% of the patients had also a history of COPD and all of them had smoking history [4-5].

In most patients, the fracture is solitary (64.3%), and Sano et al. described the right side as the most common location (57%), especially the right tenth rib (42.8%). In our case, both fractured ribs were on the left side [4].

Manual workers whose jobs involved lifting heavy weight seem to be at more risk for costal arch fracture [5]. Our patient was a retired policeman without history of weightlifting.

Conservative treatment is the first-choice approach. In cases of daily activities limiting symptoms (e.g. pain, dyspnea) or complications, such as pulmonary herniation, pneumothorax or diaphragmatic laceration, the surgical approach should be considered, as thromboembolic events are the post-operatory main complication [1-2, 5].

## Conclusions

Costal arch fractures in patients without trauma history or associated bone-disease may be missed at physical examination and plain radiography. Diagnostic delays increase the risk of complications, such as refractory pain, herniation, and organ rupture. Cough-induced rib fracture should be remembered as a possible diagnosis in patients with persistent cough and new-onset thoracic pain.
